# Multilocus Sequence Typing of *Leuconostoc mesenteroides* Strains From the Qinghai-Tibet Plateau

**DOI:** 10.3389/fmicb.2021.614286

**Published:** 2021-01-25

**Authors:** Jun Chen, Haoxin Lv, Zhixia Zhang, Hua Zhang, Bei Zhang, Xing Wang, Yuan Liu, Miao Zhang, Huili Pang, Guangyong Qin, Lei Wang, Zhongfang Tan

**Affiliations:** ^1^School of Agricultural Sciences, Zhengzhou University, Zhengzhou, China; ^2^Henan Key Laboratory of Ion-Beam Bioengineering, School of Physics, Zhengzhou University, Zhengzhou, China; ^3^School of Food Science and Technology, Henan University of Technology, Zhengzhou, China; ^4^School of Food and Biological Engineering, Henan University of Animal Husbandry and Economy, Zhengzhou, China; ^5^Department of Chemical and Environmental Engineering, Jiaozuo University, Jiaozuo, China; ^6^Academy of Animal Science and Veterinary Medicine, Qinghai University, Xining, China

**Keywords:** *Leuconostoc mesenteroides*, multilocus sequence typing, genomes, phylogenetics, housekeeping genes

## Abstract

*Leuconostoc mesenteroides* strains were a type of epiphytic bacterium widely used in fermented foods and products in the biochemical and pharmaceutical industries but data on its presence in foods from Qinghai-Tibet Plateau in China was scarce. In this study, molecular analysis based on multilocus sequence typing (MLST) with eight housekeeping genes (*pyrG, groeL, rpoB, recA, uvrC, murC*, *carB*, and *pheS*) was carried out on 45 *L. mesenteroides* strains isolated from different plants and dairy products from Qinghai-Tibet Plateau in China. The objective of this study was to perform genetic diversity analysis and explore the relationship between strains and isolate samples or separate regions. A total of 25 sequence types (STs) were identified with a diversity of up to 55.6%, which were grouped into one clonal complexes (CCs), 3 doublets and 17 singletons by eBURST. The results of minimum spanning tree and clustering analysis indicated these *L. mesenteroides* strains from the Qinghai-Tibet Plateau were relatively weakly related to the isolated region. However, there was a close relationship between the genotypes of *L. mesenteroides* strains and the type of the isolated sample, which was consistent with the results of API 50CH. The MLST scheme presented in this study provides a shareable and comparable sequence database and enhances our knowledge of the population diversity of *L. mesenteroides* strains which will be further used for the selection of industrial strains.

## Introduction

*Leuconostoc mesenteroides*, a species of *Leuconostoc*, is usually found to be associated with plant, meat, dairy, and other food products. *L. mesenteroides* is a kind of gram-positive, facultative anaerobic lactic acid bacterium usually used as starter cultures or as natural inoculum in many fermented foods such as cheese, sauerkraut, butter milk, wine, and kefir (Campedelli et al., 2015). Some studies have shown that *L. mesenteroides* plays significant roles in fermentation of foods, such as sauerkraut from Europe and kimchi from East Asia (Beganovic et al., 2011), and it contributes to the improvement of the nutritional and organoleptic properties of fermented foods. In addition, *L. mesenteroides* is also widely used in the field of in the biochemical and pharmaceutical industries (Choi et al., 2017). The demand for producing high quality dextran and their derivatives continues increasing all over the world (Naessens et al., 2005). Compared to the immense applications in pharmaceutical, food and biotechnology industries, that in public health and food safety should be paid more attention and further investigations need to cover the origin of *L. mesenteroides* because of its frequent detection in different foods and its interaction to some gastrointestinal problem in humans (Franco-Cendejas et al., 2017; Menegueti et al., 2018). In our study, Qula (a kind of fermented dairy food) is very popular in the Qinghai-Tibet Plateau region, but there are few reports about the microorganisms and food safety in Qula which poses some potential threats to diet health. Compared to other dairy production, the main component of Qula, has higher lipid, protein, lactose, and mineral levels (Zhang B. et al., 2015). In view of these excellent traits, the development of *L*. *mesenteroides* is of great potential in the application, so it is necessary to carry out diversity characterization analysis. *L. mesenteroides* species contain four subspecies: *L*. *mesenteroides* subsp. *mesenteroides*, *L*. *mesenteroides* subsp. *cremoris*, *L*. *mesenteroides* subsp. *dextranicum* and *L*. *mesenteroides* subsp. *Suionicum* (Jeon et al., 2017). The identity between the four subspecies was as high as 99%, which meant traditional molecular typing approaches such as 16s rRNA for characterization of the species at the isolate level were either unavailable or unreliable at the molecular level, and a standard protocol for their detection and characterization was not available. Only a higher resolution molecular typing technique can be used to explore its genetic evolution. Considering the important role in the fermented food industry and the production of dextrans and bacteriocins with promising developing prospect, the exploration of molecular character of *L. mesenteroides* was in great need to categorize candidate microorganisms for further safe utilization in production.

Generally, typing methods of bacteria contained phenotyping and genotyping. Phenotypic methods were traditional including serotypes, phage-types, biotypes, and antibiograms, which often brought about uncertain identification (Dan et al., 2014). Therefore, genotypic methods were playing important roles in phylogenetic classification and identification of bacterial species. Till now, many molecular methods had been used for the typing of *Leuconostoc* genus like pulsed-field gel electrophoresis (PFGE), restriction fragment length polymorphism (RFLP), amplified fragment length polymorphism (AFLP), random amplified polymorphic DNA (RAPD)-PCR, repetitive element palindromic PCR (rep-PCR), and multilocus sequence typing (MLST) (Vihavainen and Bjorkroth, 2009; Alegria et al., 2013; Sharma et al., 2018, 2020). MLST was a technique for distinguishing accurately between different isolates within a species. MLST was first proposed and applied in Maiden et al. (1998), which was a new molecular analysis method developed on the basis of Multilocus Enzyme Electrophoresis (MLEE) methods. This typing methods for intraspecies identification of pathogens were essential epidemiological tools in infection prevention and control. Shortly thereafter, MLST was applied to analyze non-pathogenic food production bacteria including LAB (Sabat et al., 2013). For example, Tanigawa and Watanabe used MLST to compare 7 housekeeping genes in 41 isolates of *Lactobacillus delbrueckii* and demonstrated MLST was efficient for identificating isolates to subspecies level (Tanigawa and Watanabe, 2011). MLST was a technique for distinguishing accurately between different isolates within a species. Compared to other methods, MLST scheme had clear results and the sequence data could be transferred worldwide with a global internet database which can realize the transmission of genetic evolution information between a wide range of strains (Olsen et al., 2014). The research field of MLST had a qualitative breakthrough until Mora promoted multilocus technology from pathogenic bacteria to non-pathogenic bacteria in Mora et al. (2000). He explored the genetic diversity of *Pediococcus acidilactic* by using MLST, finding that AcH/PA-1 pediocin produced by *Pediococcus acidilactici* had a certain relationship with its genotype (Mora et al., 2000). In Ruiz-Garbajosa et al. (2006) used MLST to amplify 110 strains of *Enterococcus faecalis*. The results showed that the genetic relationship was related with the isolated source (Ruiz-Garbajosa et al., 2006). Although the population biology of some LAB species had been characterized by MLST methods, to date, there was little MLST protocol available for *Leuconostoc* species.

In this study, based on the effective method of MLST, 41 *L. mesenteroides* strains isolated from some parts of the Qinghai-Tibet Plateau and 4 standard strains were used to explore the population structure and the relationship between the genetic relationship and the factors including the isolate region and the isolated samples. The aim of the present study was to utilize the genomic sequence to construct a multilocus sequence typing (MLST) approach for *L. mesenteroides*, which would provide a tool for characterizing isolates from Qinghai-Tibet Plateau to study globally. In addition, establishment of a sequence-based MLST library would enable global surveillance of *L. mesenteroides*.

## Materials and Methods

### Obtain of the Experimental Strains

We obtained 41 strains of *L. mesenteroides* isolated from crops and dairy products from some parts of the Qinghai-Tibet Plateau from 2008 to 2011 (Table 1), and 4 standard strains purchased from Japan and China Culture Collection Center (Table 2). All of the strains were initially identified by physiological and biochemical characteristics, including experiments of Gram reactions, catalase activity and gas production in the presence of glucose as described (Kozaki et al., 1992). Furthermore, the species were identified based on 16S rRNA gene sequence analysis with the prokaryotic 16S rDNA universal primers 27F (5′-AGAGTTTGATCCTGGCTCAG-3′) and 1492R (5′-GGTTA CCTTGTTACGACTT-3′) (Zhang et al., 2016). The PCR products were sequenced by the Huada Biotech Company (Zhengzhou, China) and subjected to BLAST analysis on the NCBI website^1^. Among these 41 strains of *L*. *mesenteroides* isolated from the Tibetan plateau, the series of qz strains represented the isolated strains naturally attached to the surface of common crops in some places of the Qinghai-Tibet Plateau, including the potato (*n* = 16), oat (*n* = 7) and wheat (*n* = 2). The series of cw strains (*n* = 16) represented strains isolated from a kind of dairy product called Qula. Stock cultures were stored at −80°C in 20% (v/v) glycerol.

**TABLE 1:** Basic information of *Leuconostoc mesenteroides*.

Strains number	Isolate sites	Isolate source	Isolate time
qz274	Near Ping-A highway in Qinghai province	potato	2011
qz275	Near Ping-A highway in Qinghai province	potato	2011
qz279	Near Ping-A highway in Qinghai province	potato	2011
qz280	Near Ping-A highway in Qinghai province	potato	2011
qz504	Huzhu country, Haidong city, Qinghai province	wheat	2011
qz534	Haiyan county, Haibei prefecture, Qinghai province	oat	2011
qz540	Near the Qinghai Lake	oat	2011
qz541	Near the Qinghai Lake	oat	2011
qz543	Huangyuan county, Xining city, Qinghai province	potato	2011
qz544	Huangyuan county, Xining city, Qinghai province	potato	2011
qz545	Huangyuan county, Xining city, Qinghai province	potato	2011
qz546	Jiangxi township, Hainan prefecture, republican county	oat	2011
qz547	Jiangxi township, Hainan prefecture, republican county	oat	2011
qz547-2	Jiangxi township, Hainan prefecture, republican county	oat	2011
qz549	Jiangxi township, Hainan prefecture, republican county	oat	2011
qz568	Huangyuan county, Xining city, Qinghai province	potato	2011
qz569	Huangyuan county, Xining city, Qinghai province	potato	2011
qz594	Huzhu country, Haidong city, Qinghai province	potato	2011
qz695	Dahua town, Huangyuan county, Xining city, Qinghai province	potato	2011
qz733	Changning town, Datong county, Xining city, Qinghai province	wheat	2011
qz768	Haiyan county, Haibei prefecture, Qinghai province	potato	2011
qz769	Haiyan county, Haibei prefecture, Qinghai province	potato	2011
qz770	Haiyan county, Haibei prefecture, Qinghai province	potato	2011
qz771	Haiyan county, Haibei prefecture, Qinghai province	potato	2011
qz772	Haiyan county, Haibei prefecture, Qinghai province	potato	2011
cw17	Guoluo Tibetan autonomous prefecture, Qinghai province	Qula	2008
cw18	Guoluo Tibetan autonomous prefecture, Qinghai province	Qula	2008
cw19	Guoluo Tibetan autonomous prefecture, Qinghai province	Qula	2008
cw20	Guoluo Tibetan autonomous prefecture, Qinghai province	Qula	2008
cw21	Gangcha county, Haibei prefecture, Qinghai province	Qula	2008
cw22	Gangcha county, Haibei prefecture, Qinghai province	Qula	2008
cw23	Huangnan Tibetan autonomous prefecture of Qinghai province	Qula	2008
cw25	Huangnan Tibetan autonomous prefecture of Qinghai province	Qula	2008
cw26	Huangnan Tibetan autonomous prefecture of Qinghai province	Qula	2008
cw27	Huangnan Tibetan autonomous prefecture of Qinghai province	Qula	2008
cw28	Gannan Tibetan autonomous prefecture, Gansu province	Qula	2008
cw29	Gannan Tibetan autonomous prefecture, Gansu province	Qula	2008
cw31	Yushu Tibetan autonomous prefecture of Qinghai province	Qula	2008
cw32	Yushu Tibetan autonomous prefecture of Qinghai province	Qula	2008
cw33	Yushu Tibetan autonomous prefecture of Qinghai province	Qula	2008
cw34	Yushu Tibetan autonomous prefecture of Qinghai province	Qula	2008

**TABLE 2:** Standard strains of *Leuconostoc mesenteroides*.

Strain	Subspecies	Time	Isolate source
JCM6124	*Leuconostoc mesenteroides* subsp. *Mesenteroides*	1980	fermenting olives
JCM16943	*Leuconostoc mesenteroides* subsp. *Cremoris*	1983	infant
CGMCC1.2141	*Leuconostoc mesenteroides* subsp. *Dextranicum*	1983	infant
CGMCC1.2138	*Leuconostoc mesenteroides* subsp. *Suionicum*	1983	fermenting olives

### Extraction and Detection of Total Genomic DNA

All of the *L. mesenteroides* strains were incubated for two consecutive generations of 24 h each in de Man, Rogosa and Sharpe (MRS) broth medium at 37°C. Total genomic DNA was extracted according to the manufacturer’s instructions in Whole Genome DNA Kit (NEP062, Beijing Dingguo, China) and the method described by Pitcher et al. (1989), and the extracted DNA was measured by a microscopic UV spectrophotometer (Thermo Fisher Scientific, Wilmington, United States) at a uniform concentration of 100 ng/μL before use. The total DNA extraction process mainly included the following steps and respective systems. lysozyme: 20 μg/ml, fully dissolved and divided into 1 ml per tube for storage in the refrigerator. Proteinase K: 20 mg/ml, stored at −20°C. RNase A: 10 mg/ml, 10 mg RNase A added to a concentration of 1 M Tris (pH 7.5) 10 μl, 2.5 M NaCl 6 μl, sterile water 984 μl, heated at 100°C for 15 min cooled and stored at −20°C. 5 × TBE Electrophoresis buffer reservoir: 54 g of Tris, 27.5 g of boric acid, 20 ml of 0.5 M/L EDTA (pH 8.0), 1000 ml of fixed volume of ultrapure water. The phylogenic tree was constructed using the neighbor-joining method with MEGA 7 software.

### Amplification and Sequencing of Housekeeping Genes

**FIGURE 1:**
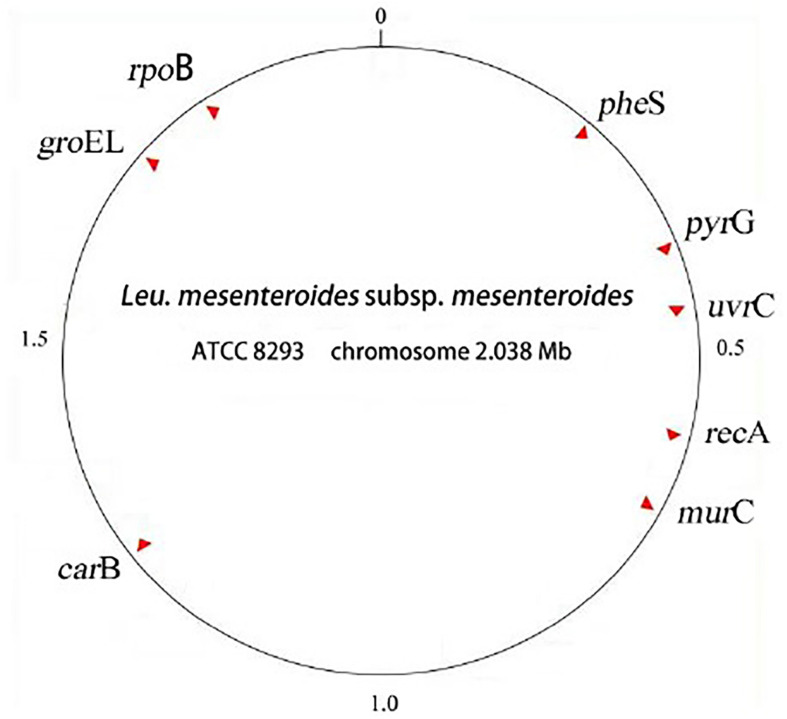
Locations of 8 MLST loci in the genome of *Leuconostoc mesenteroides* subsp. *Mesenteroides* ATCC 8293.

The selected 8 housekeeping genes were relatively evenly distributed: *pyrG*, *groeL*, *rpoB*, *recA*, *uvrC*, *murC*, *carB*, *pheS*. The distance distribution of these 8 housekeeping genes and the location of the whole genome in the reference strain *L. mesenteroides* ATCC8293 were shown in Figure 1, and the specific details were shown in Table 3.

**TABLE 3:** Genes and primers used for MLST.

Gene	Length	Gene products and functions	PCR Primer	Primer sequence	TM (°C)
*pyrG*	598	CTP synthetase	*pyrG*-F *pyrG*-R	GTTACTGGCGATGGTTTAG AGACATTTCTGCCTTTGG	55.4 52.7
*groeL*	656	chaperonin Gro EL	*groEL*-F *groEL*-R	TGAAGCCTTGCCAACA CACTCATCAGTAGCAGCGT	51.6 57.6
*rpoB*	608	DNA-directed RNA polymerase subunit beta	*rpoB*-F *rpoB-*R	GCATTTTGGAACGACTGT TGGGTTTTCGGGAGAT	52.7 51.6
*recA*	550	recombinase A	*recA*-F *recA*-R	ATCCCAAGGGGCGTAT GCAACTTTGAATGGTGGAG	54.1 55.4
*uvrC*	560	excinuclease ABC subunit C	*uvrC*-F *uvrC*-R	CTTGTTTCCGTGATGTTCC CCTTCATTGCCCCAGTC	55.4 57.0
*murC*	619	UDP-N-acetylmuramate-L-alanine ligase	*murC*-F *murC*-R	TCCATTAGAGGCAGCAGG GGTTTCAAAGAACGCAAGT	57.3 53.2
*carB*	833	carbamoyl phosphate synthase large subunit	*carB*-F *carB*-R	TAAAGCCTTGATGGAACG ATTGCGACCGATAGCC	52.7 54.1
*pheS*	665	phenylalanyl-tRNA synthetase subunit alpha	*pheS*-F *pheS*-R	AACAGGTTTGCTGAAGGG GGGAAATAAGAAGGTCGC	55.0 55.0

In the MLST scheme, the number of 7 housekeeping gene loci was the most common, and the length of the amplified fragments was concentrated around 450bp. In this experiment, in order to increase the discrimination between strains, 8 housekeeping genes were selected. The location of housekeeping genes was shown in Figure 1. It was clear that the 8 selected housekeeping genes (*pyrG, grooveL, rpoB, recA, uvrC, murC, carB, pheS*) were unlinked and evenly distributed along the genome of *L. mesenteroides* ATCC 8293, and there was a certain distance between each loci which can reflect the genetic information of *L. mesenteroides* more comprehensively (Makarova et al., 2006). The whole genome sequence size of the standard strain ATCC 8293 was 2.038 Mbp. Among them, the *pheS, pyrG, uvrC* genes were distributed at 0-0.5 Mbp, the *recA* and *murC* genes were distributed in the 0.5-1.0 Mbp interval, the *carB* gene was at 1.0-1.5 Mbp, within the range of chromosome 1.5-2.038 Mbp, *groEL* and *rpoB* genes were distributed.

The primers for the eight housekeeping genes *pyrG, groEL, rpoB, recA, uvrC, murC, carB*, and *pheS* were designed via the software Primer5.0, and then the correctness of the primers was determined by sequence alignment check. The primers used in this experiment (Table 3) were synthesized by Biotechnology (Shanghai) Co., Ltd. PCR amplifications were performed with the methods described by Lhomme et al. (2015); Sharma et al. (2018). The PCR reaction program included several steps, pre-denaturation at 94°C for 5 min, denaturation at 94°C for 1 min, annealing at 50°C for 1 min, extension at 72°C for 1 min, extension at 72°C for 10 min, in which the second step to the fourth step were repeated 35 times. The annealing temperature of *rpoB* was 48°C, and the annealing temperature of *UvrC* and *MurC* was 52°C. The PCR product was verified in 1% agarose, and after being qualified, it was sent to Shanghai Meiji Biological Company for sequencing.

### Gene Sequence Database Alignment and Analysis of MLST

The bidirectional sequence bases that had been successfully sequenced were spliced together without error, and then imported into BioNumerics V6.0 (Applied-Maths, Sint Maartens-Latem, Belgium) software to determine the sequence typing. Used the online eBURST v3.0 software^2^ to analyze the resulting STs (Francisco et al., 2009), and determined the clonal complex (Urwin and Maiden, 2003). Finally, the minimum spanning tree analysis of all STs was performed by using Prims’s algorithm in the BioNumerics software to explore the relationship between STs and different isolate regions and samples.

The Simpson Index was used to evaluate the discriminatory power of the MLST typing method established in this study for different *L. mesenteroides* strains. Simpson’s diversity index (D) was calculated according to the results of MLST (Hunter and Gaston, 1988). DNA sequence analyses were performed, including allelic variation of selected 8 housekeeping genes. The number of polymorphic sites, Tajima’s D value, nucleotide diversity (π), G + C content and the dN/dS ratio were calculated with the software DnaSp v5.1. The linkage equilibrium between alleles at the 8 housekeeping genes was detected with the program LIAN and the index of association (IA) was calculated (Murray, 2005).

### STs Clustering Analysis

The study of genetic diversity of species was based on genetic distance, which described the genetic structure and interspecies differences of a population by reflecting the phylogenetic evolutionary relationships of that population. It was generally believed that ST in populations with longer genetic distances had existed in nature for longer periods of time and have evolved in different regions. Distance-based clustering construction methods mainly included Unweighted pair-group Method Using an Arithmetic Average (UPGMA), Neighbor-joining method (NJ), and Maximum-likelihood method (ML). In this study, the UPGMA method was used to construct clustering map. An UPGMA dendrogram was constructed by START 2.0 software via UPGMA method described by Dan et al. (2014). The most prominent advantage of this method was that the results were clear, less distortion, and better representation of the affinity between different strains.

### Carbon Source Fermentation Clustering Analysis

The test was carried out using an Analytical Profile Index (API 50CH, bioMerieux, France) test strip containing 49 carbon sources and a control. The experimental method was operated according to the instructions and strains were cultivated in a constant temperature incubator at 37°C. The yellow color represents positive result, yellow-green means weak positive, and blue indicates negative. The experimental results were recorded at 24 and 48 h, respectively. Analyzed the results of the carbon source fermentation experiment, and the clustering analysis of API 50CH was performed. Furthermore, the results of the cluster analysis of carbon source fermentation experiments were compared with those of STs cluster analysis, and the relationship between the characteristics of carbon source fermentation and the STs of *L*. *mesenteroides* strains from different isolated regions and sample were discussed. It also provided a reference for the research of nutritional physiology, phylogenetic relationship and interspecies relationship of different isolated strains.

## Results and Discussion

### Sequence Alignment and Analysis of Typing Results

**FIGURE 2:**
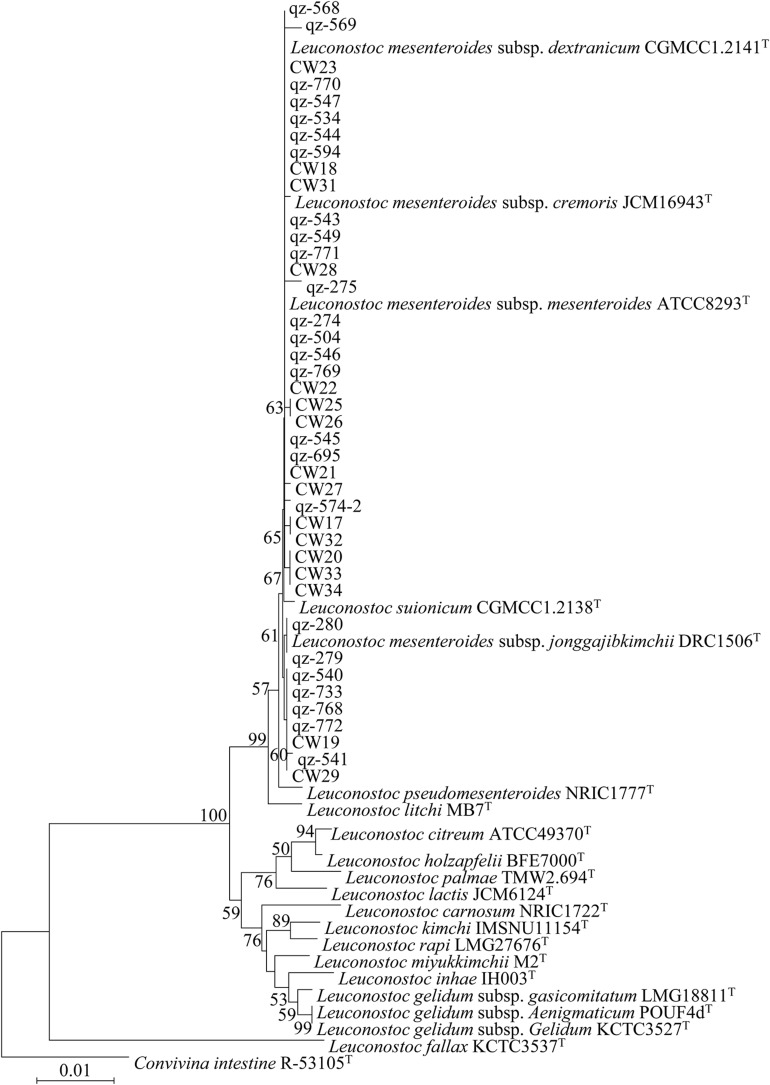
Phylogenetic trees of 45 *Leuconostoc mesenteroides* strains based on 16S rRNA gene sequences.

All 45 *L*. *mesenteroides* isolates were identified by 16S rRNA sequencing and a phylogenetic tree was constructed using the neighbor-joining method (Figure 2). They were all *L. mesenteroides*, which belong to different subspecies. In order to identify and distinguish these strains more accurately, the method of MLST was adopted. At present, there is no relevant data of *L. mesenteroides* on the PubMLST website. The eight genes (*pyrG, groeL, rpoB, recA, uvrC, murC*, *carB* and *pheS*) were successfully amplified for all 45 *L*. *mesenteroides* isolates. The sequences obtained for the eight housekeeping genes were imported into BioNumerics V6.0 software to determine the allele number. The allele numbers of each strain were linked in series in a certain order as Allelic Profiles. This set of values corresponds to a number representing the ST of the strain. The results of the typing of 45 strains were shown in Table 4. Finally, 45 *L*. *mesenteroides* were divided into 25 sequence types (STs) with a diversity of up to 55.6%. Among them, ST8, ST17, and ST19 were the most frequent, each containing 4 strains, accounting for 8.9% of all strains respectively. Next were ST9, ST10, ST13, and ST14, containing three isolated strains each, accounting for 6.7% of the total number of strains, respectively. What’s more, all of the isolated strains contained in these 4 STs were derived from crops. The remaining STs such as ST5, ST6, ST16, ST20, ST21 only had one member. The results indicated that the MLST profiles of *L*. *mesenteroides* isolates from Qinghai-Tibet Plateau in China were highly diverse. The genetic variations of the *L*. *mesenteroides* isolates for the different loci also have been shown in Table 4. Furthermore, it can be observed that the allelic frequency was dominated for alleles 1 and 2 compared to other alleles among the 8 selected housekeeping gene loci which was uniform with the results of Sharma et al. (2018). The apparent low levels of biodiversity in *groEL, recA, pheS* suggested that these gene sequences of the loci were somewhat conserved which have the lower discriminatory ability than the other housekeeping loci, and the remaining 5 loci had more polymorphic sites. Our work led to the identification of new alleles and new STs, confirming that the *L. mesenteroides* population had a great genetic diversity. Similar work on *Leuconostoc citreum* strains were performed, which also revealed the diversity of STs of 13 bacterial strains isolated from South Korea (Sharma et al., 2018). Besides, the genomic variation is associated with ecological and social interactions, and local selection pressures and population of bacteria could cause micro-evolutionary changes (Cordero and Polz, 2014). The isolated strains were examined for their genetic diversity using 7 selected seven housekeeping genes with the lower discriminatory ability rather than 8 housekeeping loci used in our study. Initially, 10 housekeeping genes were selected for MLST from the complete genome of *L*. *gelidum.* subsp. *gasicomitatum* LMG 18811^T^ in the study of Johansson et al. (2011). however three of the 10 genes including g*rpoA, dnaA* and *atpA* were deleted because they were either located too close to another gene or bring with too little variation (Rahkila et al., 2015). MLST was considered to be the most discriminating method for studying molecular epidemiology and population structure of bacteria (Baldo et al., 2006; Liang et al., 2013). Although this approach has been developed for several LAB, such as *Oenococcus oeni* (de las Rivas et al., 2004) and *Lactobacillus casei* (Diancourt et al., 2007), until this study there had been no MLST protocol used for *L*. *mesenteroides*, and the genetic diversity of this species in Chinese traditional fermented dairy food Qula had never been investigated. In this study, we used MLST with 8 housekeeping genes on 45 *L*. *mesenteroides* isolates from a large region in Qinghai-Tibet Plateau in China, making up for the blank of related research in this area.

**TABLE 4:** STs and Allelic profiles of 45 *Leuconostoc mesenteroides.*

Strain number	ST	Allele number							
		*pyrG*	*rpoB*	*groEL*	*recA*	*uvrC*	*carB*	*murC*	*pheS*
qz279	7	2	9	2	1	2	10	4	1
qz280	7	2	9	2	1	2	10	4	1
qz504	8	1	1	1	1	3	7	1	1
qz546	8	1	1	1	1	3	7	1	1
qz547	8	1	1	1	1	3	7	1	1
qz549	8	1	1	1	1	3	7	1	1
qz534	9	1	3	1	1	1	1	1	2
qz544	9	1	3	1	1	1	1	1	2
qz545	9	1	3	1	1	1	1	1	2
qz540	10	3	2	1	1	1	1	3	1
qz541	10	3	2	1	1	1	1	3	1
qz547-2	12	1	3	1	1	1	7	1	2
qz594	12	1	3	1	1	1	7	1	2
qz569	13	2	1	6	1	1	5	1	5
qz695	13	2	1	6	1	1	5	1	5
qz770	13	2	1	6	1	1	5	1	5
qz733	14	3	2	4	1	6	1	3	1
qz768	14	3	2	4	1	6	1	3	1
qz771	14	3	2	4	1	6	1	3	1
cw18	17	1	3	1	1	1	1	2	2
cw19	17	1	3	1	1	1	1	2	2
cw21	17	1	3	1	1	1	1	2	2
cw22	17	1	3	1	1	1	1	2	2
cw23	19	2	6	1	1	1	5	1	3
cw32	19	2	6	1	1	1	5	1	3
cw33	19	2	6	1	1	1	5	1	3
cw34	19	2	6	1	1	1	5	1	3
JCM6124	1	7	1	4	1	6	11	1	3
CGMCC1.2138	2	6	1	7	1	3	9	1	5
JCM16943	3	2	8	2	5	4	9	6	3
CGMCC1.2141	4	5	5	2	1	5	13	1	3
qz274	5	1	3	1	1	1	3	1	2
qz275	6	2	1	6	1	1	8	1	5
qz769	15	1	3	4	1	6	1	1	2
cw17	16	1	3	1	1	1	4	1	1
cw20	18	2	6	1	1	7	5	1	3
cw25	20	2	7	5	3	9	2	1	1
cw26	21	2	4	5	4	8	2	1	1
cw27	22	2	4	2	6	1	12	5	3
cw28	23	4	1	1	1	3	6	1	1
cw29	24	2	4	1	3	10	2	1	1
cw31	25	1	1	1	1	3	1	1	1

The D value of 0.939 calculated from Simpson’s equation indicates that the method of MLST had a high discriminatory power. Allelic variation and detection for recombination were also detected, described in Table 5. The number of alleles for each locus ranged from 5 to 13, and the number of polymorphic sites ranged from 4 to 14. The G + C content was from 37.71 to 42.58%. The average pairwise nucleotide diversity per site among the 8 genes ranged from 0.00081 to 0.00428. The dN/dS ratios of 8 loci ranged from 0.05 to 1.39. The ratio of dN/dS was less than 1 except for the gene *pheS*, indicating that external selection pressure did not interfere too much with these genes to accelerate their evolution. A similar result of dN/dS was found for the allelic variation at the MLST loci, that was, most dN/dS ratios were less than 1 except for that of *murC* (Zhang W. et al., 2015). The values from Tajima’s D test, which measures deviation from the standard neutral model of evolution, ranged from −1.72542 to 0.46268. The value of *I*_*A*_ in this study was 0.6246, indicating that the 8 selected housekeeping genes were evenly distributed throughout the genome with complete linkage equilibrium and that there was no high frequency recombination in these strains. These results above suggested balancing selection for the genes, which is typical for housekeeping genes.

**TABLE 5:** Allelic variation and nucleotide diversity of the seven MLST loci.

Gene	No. of alleles	No. of Polymorphic sites	Tajima’s *D*-value	G + C%	Nucleotide Diversity π	dN	dS	dN/dS
*PyrG*	7	7	–0.88111	37.71%	0.00319	0.00101	0.01	0.1
*rPoB*	9	8	–0.46362	38.40%	0.00428	0.00193	0.01429	0.135
*groEL*	7	5	–1.20868	41.47%	0.00105	0.00089	0.00168	0.52
*recA*	6	5	–1.72542	42.58%	0.00081	0.00014	0.00272	0.05
*uvrC*	10	9	–1.21021	39.22%	0.00243	0.00113	0.0066	0.17
*carB*	13	14	–1.09535	41.31%	0.00348	0.002	0.01399	0.14
*murC*	6	6	–1.42195	38.62%	0.00186	0.00175	0.00235	0.74
*pheS*	5	4	0.46268	38.92%	0.0027	0.00288	0.00207	1.39

### Analysis of Different STs Clonal Complexes

The online software eBURST was used to classify the MLST data of isolated representative strains and obtain non-overlapping ST groups or clonal complexes. A connection that included three and more ST types was commonly referred to as a Clonal Complex (CC) (Francisco et al., 2009). Generally speaking, the core ST of the clonal complex was the original sequence type. Although eBURST analysis was only a hypothesis of the origin and evolution relationship model of the clonal complex, it can be used to explore and analyze the diversity of bacterial clones. The eBURST aimed to identify closely related strains based on allelic profiles and is less affected by recombination than many sequence-based methods (Turner et al., 2007).

**FIGURE 3:**
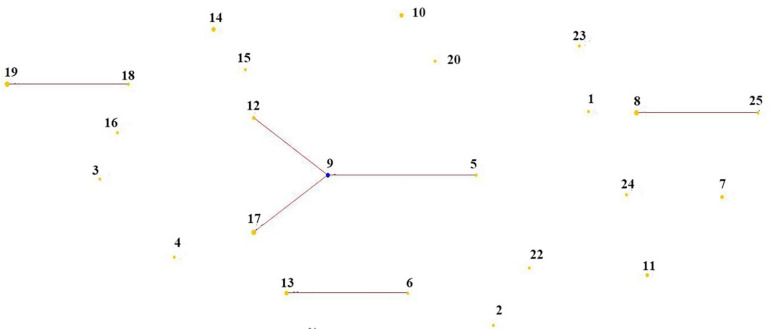
Population structure of 45 *Leuconostoc mesenteroides* strains by eBURST analysis.

According to Figure 3, 25 STs were analyzed by eBURST to eventually form a clonal complex, three doublets and 15 singletons. The clonal complex contained 4 STs. The general clonal complex was numbered by the core ST, so the core ST9 was named CC9. According to Table 4, CC9 contained a total of 10 isolates, accounting for 22.2% of the total strains. Among them, three ST types were from plants, including ST5, ST9, and ST12, while the four strains in ST17 were isolated from dairy product Qula. The Singletons contained a total of 15 ST types, containing 21 strains, accounting for 46.7% of the total and the distance between these strains is scattered, indicating that the genetic differences among the strains were relatively greater than those of other strains, possibly due to the higher conservativeness of *L*. *mesenteroides* and the lower rate of mutation of the housekeeping genes. It can also be drawn from the chart the genetic polymorphism of *L*. *mesenteroides* was high, and different strains have different evolutionary processes. The STs of strains with different isolation regions but the same kind of samples were also different, possibly due to regional environmental variation, so the strains were subjected to different selection pressures resulting in slight changes in nucleotide sequence. Similar works have been reported for *Leuconostoc lactis* isolates from traditional dairy products (Dan et al., 2014). Analysis of their MLST by eBURST produced two CCs and six singletons. The majority of *L. lactis* isolates from dairy products were found in these two CCs, while the remaining isolates from various samples including milk, pickle and yogurt were scattered into unique STs. Comparatively, 46 STs were grouped into three CCs and 17 singletons by goeBURST, and the STs in CC1 consisted mostly of strains from modified-atmosphere-packaged poultry, beef, pork, minced pork and beef (Rahkila et al., 2015). It has been reported in the previous study that usually in the genus *Leuconostoc* recombination could occur because of various mobile elements including transposable elements, bacteriophages and genomic islands (Meslier et al., 2012).

### Analysis of Genetic Evolution of ST in Different Isolation Region and Different Isolated Samples

**FIGURE 4:**
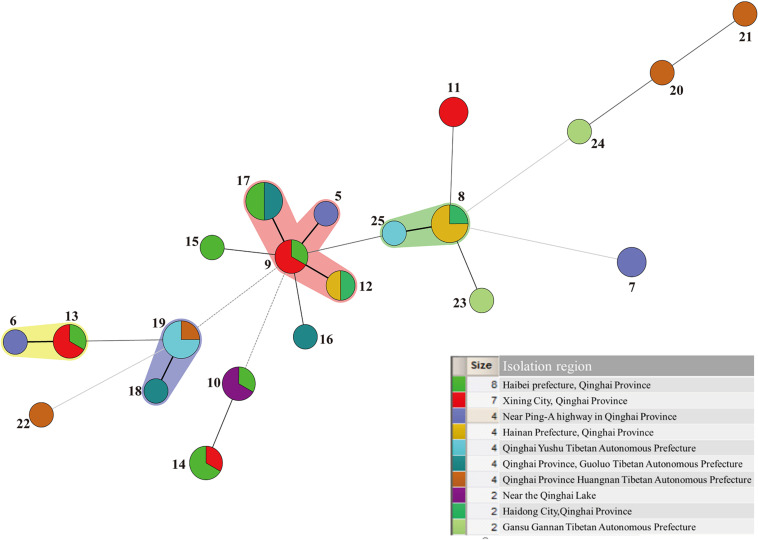
Minimum-spanning tree analysis of 45 *Leuconostoc mesenteroides* strains based on MLST date according to region. Each circle indicates a sequence type (ST), the size of the circle is proportional to the number of strains and the type of line between isolates indicates the strength of the genetic relationship between these isolates (black line = strong relationship, gray line = intermediate relationship and dotted line = weak relationship). The different colors represent the strains isolated from different region.

A minimum-spanning tree of 25 ST phenotypes was constructed using the software BioNumerics V6.0 to investigate the relationship between STs and different regions or isolated samples. The minimum spanning tree of ST of *L*. *mesenteroides* isolated from different region was shown in Figure 4. It can be seen from the figure that the ST9 core group had 4 STs, except for ST17 which contained four isolated strains from dairy products, all of which were from plants. The isolated regions of the four nearest groups of affinities were all relatively close, and their isolated strain affinities were correspondingly close. However, the different isolated strains from the same region were not all clustered together when viewed as a whole, for example, a total of eight isolated strains were isolated from Haibei State, ST9 (qz534), ST10 (qz772), ST13 (qz770), ST14 (qz768, qz771), ST15 (qz769), and ST17 (cw21, cw22), which were relatively distant from each other on the minimal generative tree, indicating their distant kinship. One of the great strengths of MLST was that it promoted the comparison of the results between different laboratories (Xu et al., 2015). In Dan’s study, clustering by region amongst the isolates was evident in the minimum-spanning tree (Dan et al., 2014). Comparable results had been reported in *Leuconostoc lactis*, where no significant associations between STs and the sources of the isolates could be found (Picozzi et al., 2010). Conclusions about the correlation between microbial diversity and geography still remain conflicting (Foschino et al., 2001; Lhomme et al., 2016; Yang et al., 2017). In the study of Yang, the genotypic diversity of *Lactobacillus sanfranciscensis* strains typed by MLST indicated geographical origin had no relation to ST of strains. In other study, the cheese isolates did not cluster together based on geographical origins, which suggesting that environmental selective pressures for strains from cheese had weaker association with geographical origin (Cai et al., 2007). The low association in *L*. *mesenteroides* strains in this study probably because of the genetic diversity of individual *L*. *mesenteroides* isolates.

**FIGURE 5:**
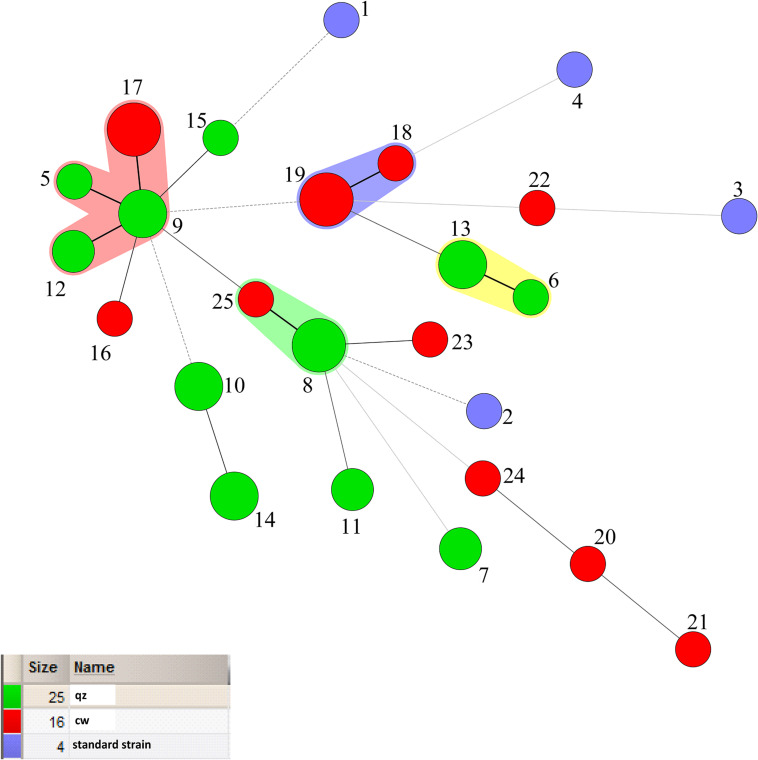
Minimum-spanning tree analysis of 45 *Leuconostoc mesenteroides* strains based on MLST date according to samples. Each circle indicates a sequence type (ST), the size of the circle is proportional to the number of strains sharing the same ST, and the type of line between isolates indicates the strength of the genetic relationship between these isolates (black line = strong relationship, gray line = intermediate relationship and dotted line = weak relationship). The different colors represent the strains isolated from different samples.

The minimum spanning Tree of STs for the different isolated samples was shown in Figure 5. Red color indicated isolated samples from oats, wheat, potatoes, while green color indicated isolated samples from dairy Qula and 4 blue circles were standard strains. Besides, Figure 5 also had four shaded areas, and the STs grouping with the closest kinship was consistent. However, the population structure of the isolated sample group had a more obvious correlation than the population structure of the isolated region. Interestingly, the strains contained in pink shaded area were all derived from plants except ST17 which came from Qula. It was found that most of the solid lines were connected to the same isolated sample except for the green shaded area, ST25 from dairy products and ST8 from plants. In addition, the strains contained in the other shaded areas were all the same source of isolated samples. The standard strain had a far-reaching relationship with the experimental strain. The whole picture showed that the relationship between the strains was more closely related to the isolated samples. Previous studies on the population genetics of food-associated bacterial species indicated that the division into subpopulations reflects adaptations to different niches rather than geographical isolation (Bridier et al., 2010). However, different results have been reported that the evolution of different *Leuconostoc* strains was not related to respective food sources (Sharma et al., 2018).

### Clustering Analysis of STs

**FIGURE 6:**
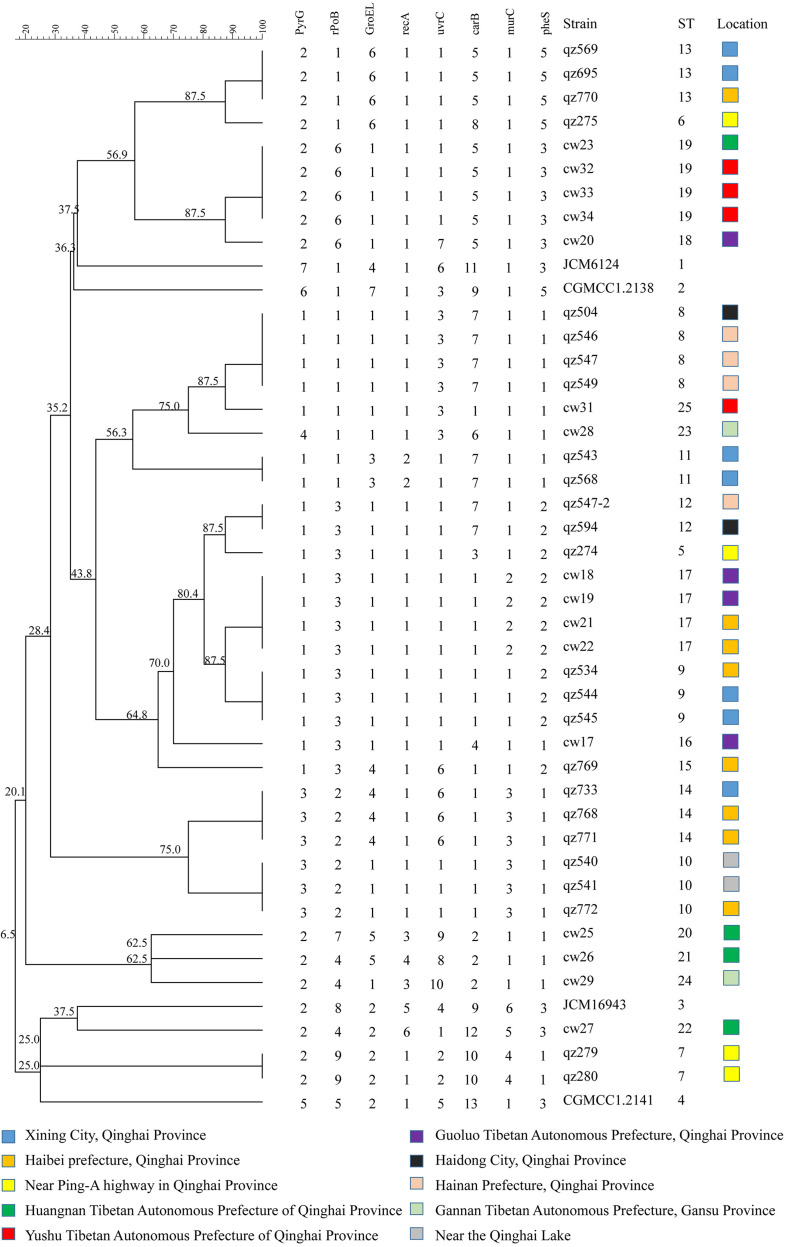
An UPGMA dendrogram of the ST of 45 *Leuconostoc mesenteroides* strains. The different colors represent the strains isolated from different location. Each row shows in turn the number of alleles, ST and isolated location for each strain.

In this study, the similarity coefficient was calculated by BioNumerics V6.0 software using the categorical algorithm, and a cluster diagram of 45 strains (25 STs) was constructed using UPGMA method, as shown in Figure 6. The characterization of genetic diversity was also reflected in the UPGMA dendrogram. The four standard strains had further genetic distance with other strains, which is consistent with the theory that genetic distances exist in nature for a long time. The branches of ST1 and ST2 were relatively close, suggesting the information of the constructed cluster graph was reliable. Most of the STs were showing some degree of relationship. There were 4 clusters in the branches with confidence above 80%, which were the first group ST6 and ST13 each with 4 strains respectively; ST18 and ST19 in second group were all from Qula; ST8 and ST25 in the third group contained 9 strains and in the fourth group consisted of 10 strains. These four groups were all on the same branch, and the genetic distance was close, indicating that the group differentiation time was short and the relationship was close, which was consistent with the results of the four shaded areas of the minimum spanning tree. Interestingly, the most of Qula-derived *L*. *mesenteroides* were clustered together, suggesting that they may have a common recent ancestor, despite their different geographical locations. The other genetic distances were relatively far, indicating these strains may have undergone some variation during the evolution process. Similar work had been reported for the UPGMA tree based on the MLST scheme. No clear link existed between ST and the original source of each isolate (Dan et al., 2014). There was no significant relationship between STs and the various sources of the strains in *L. sanfranciscensis* (Picozzi et al., 2010). The absence of association in *L. lactis* may due to the genetic diversity of strains (Passerini et al., 2010). It was found that the evolution of different *Leuconostoc* strains was not related to respective food sources (Sharma et al., 2018). A systematic and detailed study on the MLST scheme of *L*. *mesenteroides* has been reported back in Zhang W. et al. (2015). In comparison, our strains come from a wider variety of sources, not only from dairy products, but also from agricultural crops. The differences in selected housekeeping genes and the strain source, including the geographical location of the strains and the source of the isolated samples may have contributed to the different MLST results.

### Clustering Analysis of Carbon Source Fermentation

**FIGURE 7:**
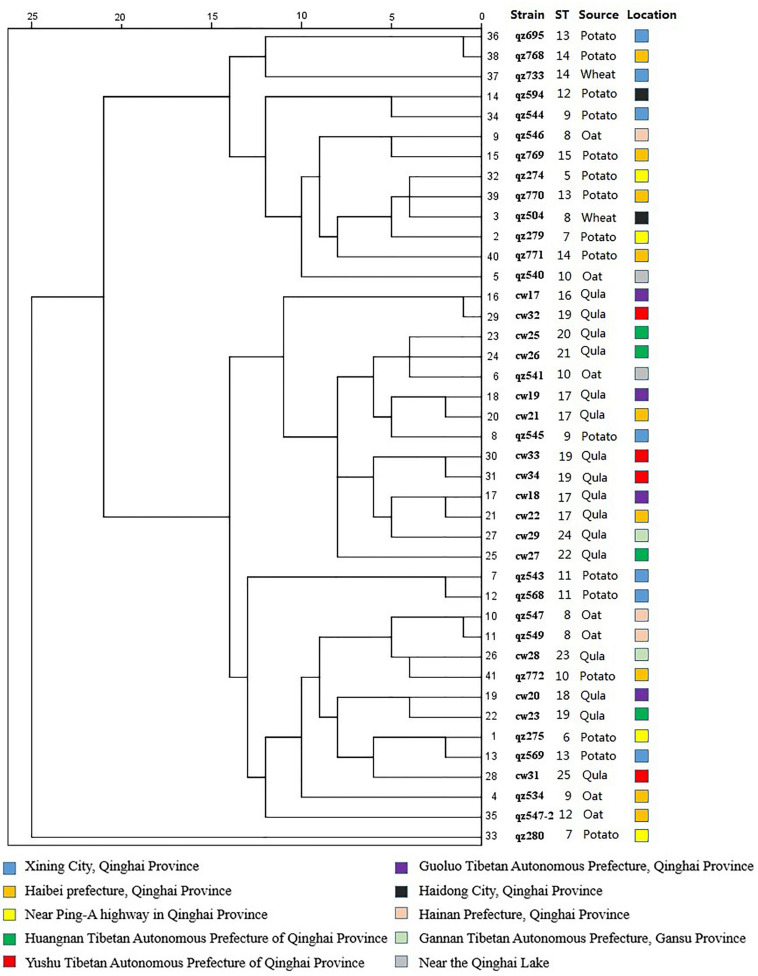
Clustering analysis of carbon source fermentation of *Leuconostoc mesenteroides*. The different colors represent the strains isolated from different location.

The results of carbon source fermentation characteristics of *L*. *mesenteroides* isolated from oat, wheat, potato and Qula showed most of the strains from the same kinds of samples had a certain similarity in carbon source utilization capacity. Clustering analysis can classify bacteria with similar carbon source metabolism characteristics, thus effectively reflecting the metabolic relationship of different strains. Taking the utilization of 49 carbon sources as clustering variables, the clustering analysis was performed using the system clustering function in SPSS software. The results, reported in Figure 7, showed that the sorts of samples clustering was more obvious, indicating the correlation was greater, while the region clustering was not obvious, which was consistent with the results of the ST clustering analysis. The carbon source fermentation results of 16 strains of *L*. *mesenteroides* from Qula were relatively close, so the clustering distance is closer, as shown, cw17, cw32, cw25, cw26, cw19, cw21, cw33, cw34, cw18, cw22, cw29, cw27 were clustered together, and they were all derived from the sample of Qula, and this group of similar carbon fermentation characteristics also included qz541 from oats and qz545 from potatoes. It was found that in ST clustering analysis in Figure 6 cw17, cw18, cw19, cw21, cw22, qz545 were also clustered together, which suggested that for strains from the same isolated samples, not only STs, but also carbon source fermentation characteristics may cluster together. In addition, we found that some strains with the same ST were similar in the carbon source fermentation clustering, for example, the strains of qz768, qz733, qz771 were all belong to ST14, and their carbon source fermentation results were clustered together, similar groups were ST8, ST11, ST17, and ST19. In a word, there may be a relationship between the ST and carbon source fermentation characteristics of *L. mesenteroides*. Other surveys had similarly found, Lactose utilization was less prevalent in plant isolates than in those from cheese and human GI tracts, probably due to relatively recent acquisitions of lactose metabolic genes (Cai et al., 2007). However, there was no significant relationship between the location of the isolated strains and the carbon source fermentation characteristics compared to the sorts of isolated sample of strains, which was similar with the results of ST clustering analysis. Moreover, it was reported the metabolic properties were slightly different because of microbial evolutionary adaption to the environment (Lee and Chang, 2016). The metabolic fingerprinting of *Arcobacter butzleri* resulted less discriminatory than the genome-based approach (Fanelli et al., 2020). Different results was reported that the metabolic properties of strains were not correlated with the two different source of isolation from shellfish (Fanelli et al., 2019). Furthermore, compared to previous studies on MLST scheme of *L*. *mesenteroides* (Zhang W. et al., 2015), phenotypic studies of carbon source fermentation and genotypic studies of MLST were combined for the first time, revealing the existence of a link between them. Many *L*. *mesenteroides* strains isolated from the same sample, their carbon source fermentation results clustered together, and some strains with the same ST also clustered together (Figure 7). The results tentatively confirmed that there was a link between genotype and phenotype, which deserved further in-depth study.

## Conclusion

The presented MLST scheme was found to be a robust tool for the investigating the population structure of *L*. *mesenteroides* from Qinghai-Tibet Plateau. A total of 25 STs were generated from 45 strains of *L*. *mesenteroides* with a diversity of 55.6%. 25 STs were analyzed by eBURST to form a clonal complex CC9, three doublets and 15 singletons. The methods of minimum spanning tree and ST clustering analysis were used to explore the relationship between strains of *L*. *mesenteroides* from Qinghai-Tibet Plateau. It was found that STs of strains from a region did not show obvious aggregation, indicating the correlation between the strains and the isolated region was relatively weak. Specifically, the STs did not cluster significantly according to the isolated regions of the strains in either the minimum spanning tree analysis or in the STs clustering analysis. However, there was a more obvious relevance between the relationship and the types of the isolated samples, that is, the representative strains of the same type of samples were more closely related, and their STs had a tendency to cluster together. The results of clustering analysis of STs and carbon source fermentation showed that there was a certain correlation between the ST and types of isolated sample and the carbon source fermentation characteristics of these strains. This is because we found that the carbon source fermentation results of strains from the same isolated samples had a tendency to cluster together, especially for strains isolated from Qula, and there were some strains with the same ST whose carbon source fermentation results also clustered together. This study revealed the diversity of *L*. *mesenteroides* from the Qinghai-Tibet Plateau and supplemented the relevant information about *L*. *mesenteroides* in the MLST online database, and provided certain contribution and clues to the molecular evolution and prevalence of this species. Further work will be focused on the biodiversity, clonal population structure and genetic recombination to obtain a better understanding of the evolution and population genetics of *L*. *mesenteroides.*

## Data Availability Statement

The raw data supporting the conclusions of this article will be made available by the authors, without undue reservation.

## Author Contributions

JC, ZZ, and ZT designed the study and wrote the manuscript. JC, HL, and ZZ performed the experiments. JC and ZZ conducted the statistical and bioinformatics analysis. LW, HZ, BZ, and YL performed the analysis of some of the data. All the authors reviewed and approved the final version of the manuscript.

## Conflict of Interest

The authors declare that the research was conducted in the absence of any commercial or financial relationships that could be construed as a potential conflict of interest.

## Footnotes

1 http://www.ncbi.nlm.nih.gov

2 http://eburst.mlst.net
